# Bone Mass, Microarchitecture, and Morphometric Insights on a Right Unilateral Bifid Mandibular Condyle: A Micro-CT Analysis Report and Literature Review

**DOI:** 10.3390/diagnostics15192440

**Published:** 2025-09-25

**Authors:** Carlos Torres-Villar, Juan Pacheco Muñoz, Eva Maranillo, Nicolás E. Ottone

**Affiliations:** 1 Departamento de Ciencias Morfológicas, Facultad de Ciencias, Universidad San Sebastián, Puerto Montt 5501842, Chile; juan.pacheco@uss.cl; 2 Doctoral Program in Morphological Sciences, Universidad de La Frontera, Temuco 4780000, Chile; 3 Department of Human Anatomy and Embryology, Complutense University of Madrid, 28040 Madrid, Spain; evamaranillo@med.ucm.es; 4 Laboratory of Plastination and Anatomical Techniques, Universidad de La Frontera, Temuco 4780000, Chile; 5 Adults Integral Dentistry Department, Center for Research in Dental Sciences (CICO), Faculty of Dentistry, Universidad de La Frontera, Temuco 4780000, Chile; 6 Center of Excellence in Morphological and Surgical Studies (CEMyQ), Universidad de La Frontera, Temuco 4780000, Chile

**Keywords:** bifid mandibular condyle, anatomical variation, temporomandibular joint

## Abstract

**Background/Objectives:** The bifid mandibular condyle (BMC) is a rare anatomical variation characterized by a division of the mandibular condyle into two distinct heads. Although frequently identified through radiographic studies or in dry skulls, its etiology remains unclear, and few studies have examined its internal bone structure. This study aimed to perform a detailed morphologic and microarchitectural analysis of a right unilateral bifid mandibular condyle using micro-CT and to contrast the findings with the relevant morphological and clinical literature. **Case Presentation:** A human mandible from an anatomical collection was analyzed. The mandible was scanned using a Bruker 1273 micro-CT system, and a 3D reconstruction was performed. Morphometric analysis was carried out on both the bifid right and normal left condyles, evaluating cortical and trabecular components separately. Parameters included bone volume, absolute bone volume, bone surface, trabecular thickness, separation, and number. The right condyle was divided into medial and lateral heads with independent necks, displaying asymmetry in size and shape. Micro-CT revealed reduced cortical volume and greater trabecular separation in the BMC, suggesting lower bone density compared to the left condyle. **Conclusions:** This case reveals significant differences in bone architecture between the BMC and the contralateral condyle, indicating a potentially reduced biomechanical capacity on the affected side. These findings emphasize the importance of incorporating microstructural evaluation in anatomical and clinical assessments of BMCs and provide novel insights that may inform diagnosis, treatment planning, and understanding of temporomandibular joint disorders.

## 1. Introduction

The bifid mandibular condyle (BMC) is a rare anatomical anomaly characterized by a division within the mandibular condyle, the portion of the jaw that articulates with the skull at the temporomandibular joint. This phenomenon, although uncommon, is of great interest in the disciplines of dentistry, maxillofacial surgery, and radiology due to its clinical relevance in mandibular procedures, including implant surgery and its distinctive radiographic presentation [1,2]. The term bifid comes from the Latin *bifidus*, which means split, divided, into two parts, and in this sense, split refers to an opening that does not completely divide a specific structure. However, the condyle’s division level is variable and can range from a shallow groove to the presence of two entirely separate condyles, each with its neck [3]. In the context of the condylar process, this implies that the normally unitary structure of the mandibular condyle is separated into two heads or portions, which may be of various sizes and shapes. This condition can occur unilaterally, affecting only one side of the jaw or bilaterally, involving both condyles [3].

Although the first description of the bifid condylar process is generally attributed to Hrdlička (1941) [4], in fact, Le Double (1906) had already documented this anomaly in 1906 [5]. However, the term “bifid condyle” or “double” was not explicitly used to refer to this structure: “Instead of the entire shape of the mandibular condyle being modified, it may only be that of one or more of its parts. Thus, its articular part can be divided into two triangles with a lower vertex, where the external triangle is usually smaller than the internal one, separated by the sharp edge of the sigmoid notch, extended excessively backward”.

In the living population, Schier 1948 described a BMC for the first time in a living person [6]; it is uncommon, it alters the morphology of the temporomandibular joint (TMJ), and there may be three or four condylar lobes [1,7].

Theories regarding the etiology of the bifid condylar process include both congenital and acquired factors. It has been suggested that it could be the result of an anomaly in the embryonic development of condylar cartilage, such as the persistence of transient embryological structures, including vascular channels or septa [8], which may lead to abnormal cartilage formation. Additionally, some authors propose that BMCs may develop as a sequela of trauma or infection in the condylar region during childhood, potentially disrupting the normal development of the mandibular condyle [9,10]. In this regard, several reports have associated the prevalence of BMCs with TMJ trauma [11,12]. Likewise, Antoniades et al. (2004) identify the alteration in the bone remodeling process in patients with BMC [13]. Additionally, some research has associated the bifid condylar process with certain genetic conditions and syndromes [14]. Overall, the lack of definitive evidence concerning its origin suggests that the etiology of the BMC remains uncertain and likely multifactorial.

The prevalence of BMCs is low and variable, from 0.18% to 12.4% (Table 3). Although they can present without symptoms (40.6%, according to Borrás-Ferreres et al., 2018) [9], in some instances, they can manifest with hypomobility, arthralgia, joint noise, and ankylosis [9,15]. This was also previously reported by Nikolova et al. (2017) [8], who also indicated that, although a BMC is not directly linked to symptoms of the temporomandibular joint, its presence is also associated with the presence of pain, trismus, and facial asymmetries, which could determine the need for surgical intervention. However, in these cases where patients present symptoms, they may be like those of other temporomandibular dysfunctions, making the diagnosis difficult [16].

Imaging plays a fundamental role in the diagnosis of the bifid condylar process. Panoramic radiographs are usually the first diagnostic tool used, although computed tomography offers a more detailed view of the condylar structure, enabling three-dimensional visualization of the anomaly [2]. In this context, the number of reported cases has increased in recent years, likely due to the growing use of imaging techniques—such as radiographs and computed tomography—for both diagnostic evaluation and surgical planning [8]. Most descriptions of BMCs in the international literature correspond to radiological findings from orthopantomography and magnetic resonance studies [15].

While these imaging modalities are effective in detecting morphological alterations, they offer limited insight into the internal bone architecture of the condyle. Given the functional relevance of the mandibular condyle in load transmission, a deeper understanding of its internal microarchitecture is essential. Micro-computed tomography (micro-CT) allows for high-resolution analysis of trabecular bone parameters such as thickness, separation, number, and connectivity, which can reflect adaptive remodeling or pathological processes. In the context of BMCs, these structural characteristics remain largely underexplored but may have important implications for joint function, degenerative changes, or surgical planning.

Although the literature reports on the bifid condylar process from imaging findings or dried samples, its exact etiology is not completely understood, and there are no details on the structural morphology of the BMC. Due to the above, the objective of this study was to carry out an osteological description of a right unilateral bifid condylar process and determine the mass and bone microarchitecture from an imaging analysis with micro-CT and contextualize the findings through a review of existing literature on this anatomical variant.

## 2. Case Presentation and Micro-CT Analysis Report

The present study was conducted on a dry human mandible obtained from the anatomical collection of the Department of Morphological Sciences at Universidad San Sebastián (Puerto Montt, Chile). The sex and age of the person it belonged to was unknown. The specimen formed part of the institution’s teaching and research resources and was preserved in compliance with Article 146 of the Chilean Health Code, which governs the ethical use of human cadaveric material for scientific and educational purposes in Chile.

The mandible was photographed with a Sony A6000 APSC camera with a Sigma (Kawasaki-shi, Japan) 30 mm F1.4 lens at 1/60 s and ƒ/16 and scanned on a micro-CT 1273, Bruker (Billerica, MA, USA). The following parameters were used for scanning: 1 mm aluminum filter, 100 keV, 145 mA, 50 µm resolution, exposure = 455 ms, rotation = 180°, and rotation step = 0.35°. The voxel size was 50 µm^3^. Image segmentation and binarization were performed using the Otsu thresholding method after histogram inspection. A 3D Gaussian smoothing filter was applied prior to segmentation to enhance image clarity and reduce noise.

The mandible was oriented in a standard anatomical position, with the condylar heads facing upward during scanning. Scanning and analysis were performed using Bruker’s micro-CT 1273 system and CTAn (v1.18.8.0), CTVox (v3.3.1), and CTVol (v2.3.2.0) software. Regions of interest (ROIs) were defined at three standardized axial levels (superior, middle, and inferior) in the condylar head. Reconstructed images were inspected for consistency, and binarization thresholds were cross-validated between left and right condyles to minimize segmentation bias.

To validate segmentation accuracy, a subset of volumes was manually segmented and compared with automated outputs. Although calibration phantoms were not available for this cadaveric specimen, internal consistency was assessed through contralateral comparison. Intra-observer repeatability was evaluated by reprocessing 20% of the volumes two weeks apart, yielding a coefficient of variation below 5% across all morphometric parameters

**Figure 1:**
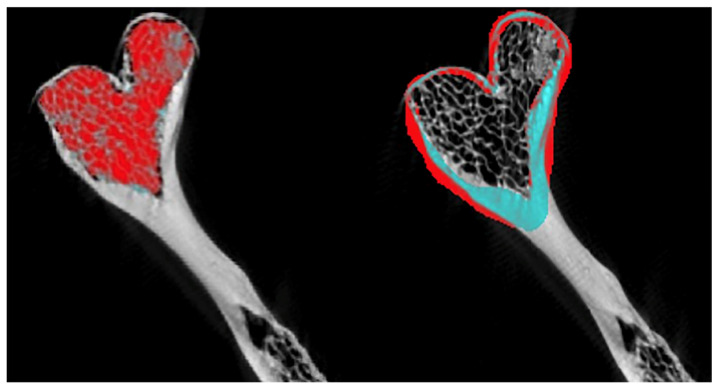
Region of interest (ROI) in cortical and trabecular bone of the BMC using CTAn. Left ROI for trabecular bone and Right ROI for cortical bone.

Using the CTAn software (v. 1.15), a 3D reconstruction was carried out from 2D images stored in .bmp format with a gray scale ranging from 0 (black) to 255 (white). This reconstruction was obtained using a surface representation algorithm from 2D images. The morphometric analysis was performed with a volume of interest (VOI) located in the right and left condylar processes, establishing circles in the initial, intermediate, and final sections. Cortical and trabecular bone were analyzed separately (Figure 1).

To determine the bone mass and microarchitecture of the mandibular condyles, coronal slices were made at the level of the mandibular rami with the CTVox software (v. 2.5), and the following basic morphometric parameters were considered [17,18,19]:

1. Bone volume (BV): the amount of space occupied by bone tissue within the volume of interest (mm^3^).
2. Absolute bone volume (C.BV/C.TV, in %): represents the cortical and trabecular bone volume within the volume of interest.
3. Bone surface (BS): total area of the bone surfaces within the volume of interest (mm^2^).
4. Mean trabecular thickness (Tb.Th, in µm): mean thickness of the trabecular structure according to a spherical fit method.
5. Mean trabecular separation (Tb.Sp, in µm): mean thickness of the void space in a trabecular structure according to a spherical fit method.
6. Trabecular number (Tb.N, in mm): number of transversals along the structure.

Related to the human sample, the specimen is stored in the anatomical collection at Universidad San Sebastián in Puerto Montt, Chile, in accordance with Article 146 of the Chilean Health Code. As the authors of this scientific work, we extend our deepest gratitude to those who selflessly donated their bodies to science, as well as to their families. We hold the utmost respect for their invaluable contributions, which not only advance anatomical research and enrich our collective understanding but also significantly enhance clinical, imaging, and surgical practices for the benefit of patients [20]. Additionally, we emphasize that the inclusion of images derived from these donations further supports the dissemination of anatomical knowledge, fostering progress in both education and healthcare [21].

### 2.1. Right Bifid Mandibular Condyle

**Figure 2:**
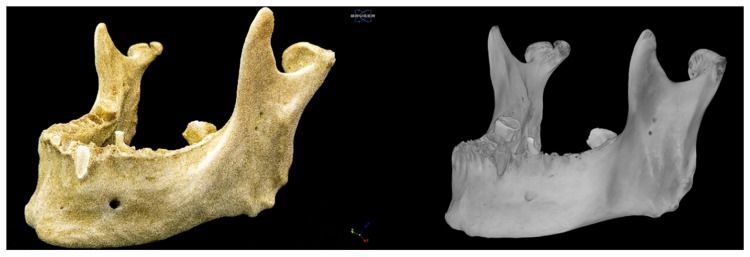
Left anterolateral view of the mandible.

**Figure 3:**
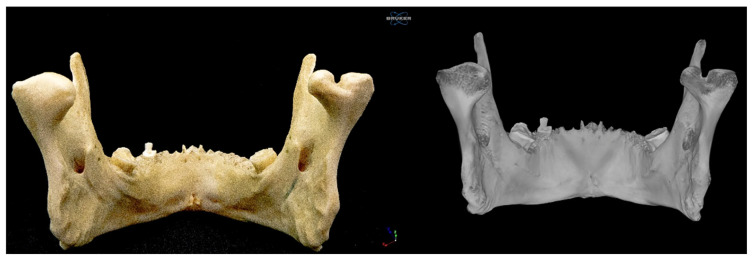
Posterior view of the mandible.

The proper condylar process was divided into two condyles, medial and lateral, by a groove/incisura 5.23 mm deep and 6.78 mm long, which followed an anteroposterior path parallel to the median plane. The medial condyle was more rounded and smaller than the lateral condyle. Its major (lateromedial) and minor (anteroposterior) axes measured 11.89 mm and 10.06 mm, respectively. It should be noted that this condyle was provided with a neck, which followed an oblique path in an anteromedial direction and was independent of the neck of the lateral condyle. The length and thickness of the neck were 8.23 mm and 10.08 mm, respectively (Figure 2 and Figure 3).

**Figure 4:**
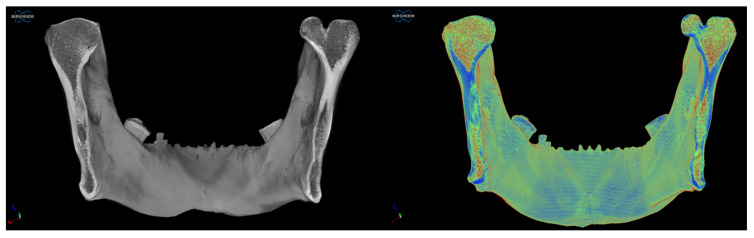
Comparison of two coronal sections at the level of the mandibular rami. Greater cortical bone is observed in the middle third of the mandibular rami.

**Figure 5:**
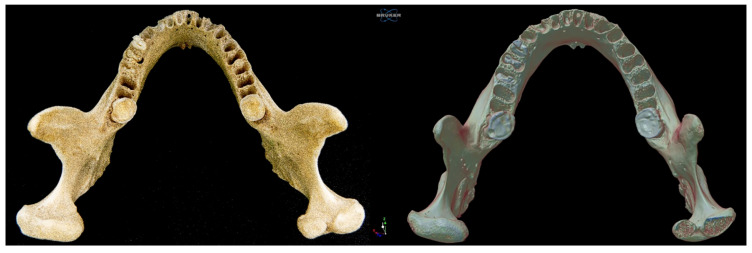
Superior view of the mandible. Note that in the BMC scanned by micro-CT, a horizontal cut was made in the BMC to appreciate the cortical and trabecular bone.

The larger lateral condyle was kidney shaped. Its major and minor axes measured 16.76 mm and 10.57 mm, respectively (Figure 1, Figure 2, Figure 3, Figure 4 and Figure 5). The neck of the lateral condyle was wider (18.92 mm) and shorter (8.84 mm) than that of the medial condyle, and it was aligned vertically with the ramus of the mandible (Figure 4 and Figure 5).

### 2.2. Mass and Bone Microarchitecture of the Mandibular Condyles

Through coronal slices at the level of the mandibular ramus, a more significant amount of cortical bone could be seen macroscopically at the level of the middle third of the rami, with a predominance on the left side. The trabecular bone had a more significant presence at the level of the mandibular condyles and in the lower third of the rami, at the level of the masseteric and pterygoid tuberosities (Figure 4).

Regarding the morphometric parameters analyzed, in the right BMC, the bone volume and absolute bone volume were 727.46 mm^3^ and 413.27 mm^3^ or 64.70% and 35.91% in the cortical and trabecular bone, respectively. The bone surface was 2191.93 mm^2^ and 3189.51 mm^2^; the mean trabecular thickness was 1281 and 721 µm; the mean trabecular separation was 648 and 805; and the trabecular number was 504 and 497 mm^−1^ in the cortical and trabecular bone, respectively.

In the left MC, the bone volume and absolute bone volume were 1291.68 mm^3^ and 444.63 mm^3^ or 71.81% and 31.21% in the cortical and trabecular bone, respectively. The bone surface was 3120.84 mm^2^ and 4239.31 mm^2^; the mean trabecular thickness was 1690 and 440 µm; the mean trabecular separation was 581 and 808; and the trabecular number was 422 and 694 mm^−1^ in the cortical and trabecular bone, respectively. Quantitative values for bone morphometry are presented in Table 1.

### 2.3. Limitations

The age, sex, and clinical history of the donor were unknown, which limited the extrapolation of biomechanical or clinical conclusions. Key factors such as skeletal maturity, edentulism, systemic bone conditions, and functional status can significantly influence mandibular bone architecture. Moreover, due to the rarity of this anatomical variant and the constraints of available material, only one specimen was analyzed. As a result, this study did not attempt to establish direct clinical correlations. Instead, the focus was placed on anatomical and microstructural characterization of the BMC, which may hold clinical relevance but should be interpreted with caution. Any clinical implications discussed are speculative, based solely on structural observations and the available literature. Expanding this research to include a larger number of specimens will be essential to determine whether the observed microarchitectural features represent consistent patterns or case-specific variations.

## 3. Discussion

**Overview and Historical Context**

The literature on bifid mandibular condyles (BMCs) presents a complex yet increasingly understood anatomical variation, with significant contributions from both historical anatomical studies and modern radiographic analyses. Historical studies, such as those by Le Double (1906) [5], Hrdlička (1941) [4], and Nikolova et al. (2017) [8], provided foundational insights into BMCs in dry human mandibles, emphasizing unilaterality and early structural observations. Le Double suggested that the alteration may not involve the entire mandibular condyle but rather just one or more of its components. Hrdlička described “double condyles” in 21 human jaws and one gorilla from the Smithsonian Institution’s collection, noting unilateral cases as more common and a slight female predominance. He also reported arthritic changes in the joint. Nikolova et al., analyzing 500 male dry jaws from the National Museum of Military History of Bulgaria, identified four unilateral BMCs (0.8%), two on each side, with no bilateral cases and no evidence of trauma. In all samples, the division of the mandibular condyle was found in the sagittal plane through a fissure of variable depth, determining the appearance of a lateral and a medial head. Notably, no traumatic damage was observed that could account for the origin of the BMC [8]. More recent cone beam computed tomography (CBCT)-based investigations, including those by Gupta et al. (2022) [22] and Yelken Kendirci et al. (2023) [23], have revealed higher prevalence rates and more nuanced morphological variations, such as trifid and tetraphid condyles. Integrating the findings from individual case reports (Table 2) and large-scale prevalence studies (Table 3) therefore offers a comprehensive perspective on the frequency, distribution, structural complexity, and biomechanical implications of BMCs across both historical and contemporary contexts. Our case contributes to this evolving body of knowledge by providing, to our knowledge, the first detailed microarchitectural analysis of a dry human bifid condyle using high-resolution micro-CT, enabling a unique quantitative comparison with the contralateral condyle and revealing structural differences that may support developmental hypotheses.

**Case Reports and Proportional Analysis (Table 2)**

Table 2 presents a review of 66 reported cases of bifid mandibular condyles (BMCs) documented in the literature, categorized by sex and side. The distribution is evenly split between males (33 cases) and females (33 cases), indicating no significant sex predilection. Regarding laterality, unilateral cases predominate, comprising 91% of the total (26 left-sided and 34 right-sided), while bilateral occurrences account for 9% (6 cases). Among unilateral cases, the right condyle appears to be slightly more frequently affected than the left. Notably, the study by Gupta et al. (2022) [22] stands out with the highest number of cases (40 in total), contributing approximately 33% of all reports included in the table. A clear temporal trend is observed, with a marked increase in reported cases from the early 2000s, likely reflecting the expanded use of advanced imaging techniques, such as CT and CBCT, as well as a heightened interest in anatomical variations. In contrast, earlier reports from the 1980s and 1990s [44,45] typically describe isolated cases, suggesting either lower detection rates or underreporting in that period. Overall, the data suggest that the BMC is a rare anatomical variant, predominantly unilateral, with a nearly equal sex distribution and a slightly higher frequency on the right side. In alignment with these findings, the bifid condyle analyzed in our study was also located on the right side and presented as an isolated, unilateral variant in a dry human mandible. Its microarchitectural assessment adds a novel quantitative dimension to the current literature, which has so far primarily focused on morphological and prevalence data.

**Prevalence in Imaging Studies (Table 3)**

The reported prevalence of bifid mandibular condyles (BMCs) in the literature varies widely, ranging from as low as 0.11% to as high as 12.4%, depending on the population studied and the diagnostic methods used. Studies based on large samples, such as those by Menezes et al. (2008) [14] and Miloglu et al. (2010) [39], report low prevalence rates (below 1%), whereas smaller studies, such as those by Balaji (2010) [38] and Khojastepour et al. (2015) [42], report substantially higher rates, suggesting potential sampling bias or regional anatomical variation. More recent investigations, like that of Gupta et al. (2022) [22], report intermediate prevalence values (4.7% per patient and 2.3% per condyle), likely reflecting advances in diagnostic imaging, particularly the growing use of CBCT. In terms of sex distribution, most studies that differentiate by sex show slightly higher or comparable prevalence in males compared to females. For example, Gupta et al. [22] reported 5.6% in men and 4% in women, while Gündüz et al. (2015) [43] found 0.72% in men and 1% in women. Lateral distribution of BMCs shows a slightly higher frequency on the right side, as seen in Miloglu et al. [39] (0.14% right vs. 0.1% left) and Gupta et al. [22] (1.6% right vs. 1.2% left). Bilateral cases remain relatively uncommon across all studies, with the highest recorded prevalence being 3.3% (Balaji, 2010) [38]. It is important to note that several authors recorded bilateral BMCs as single cases rather than two condyles, which may lead to underestimation of total condylar involvement. Additionally, not all studies provided sex- or side-specific data, limiting comprehensive comparisons. Overall, the BMC appears as a relatively rare anatomical variation with a slight male and right-side predominance, and its reported prevalence has increased in recent years likely due to improved imaging modalities and greater clinical awareness. Our case, which involved a unilateral right-sided bifid mandibular condyle identified in a dry human mandible, aligns with the most commonly reported patterns in the literature. Although prevalence cannot be inferred from isolated anatomical specimens, the morphological characteristics observed are consistent with those described in population-based imaging studies, further supporting the relevance of individual case analyses in enriching our understanding of this variation.

**Sex Distribution and Methodological Considerations**

Sex-specific prevalence data in Table 3 are inconsistently reported. Where available, data from Gupta et al. (2022) [22] and Gündüz et al. (2015) [43] show slightly higher rates in males, noting a reversal with higher rates in females. The lack of consistent reporting for bilateral cases further complicates accurate sex-based comparisons. Despite this, the data from both tables align on several points: BMC is predominantly unilateral, lacks a strong sex predilection, and appears slightly more frequently on the right side. The increasing detection in recent decades underscores the importance of advanced imaging in identifying this anatomical variant. Additionally, the observed inconsistency in reporting sex-specific and laterality data across studies highlights a broader methodological issue. Many articles report only raw case distributions without contextualizing them within the total population analyzed by sex or side. This results in the inability to calculate actual prevalence rates for subgroups such as males, females, left or right condyles. As described in the introduction, this ambiguity affects our understanding of the true sex-related distribution and the biological or developmental factors that may underlie these anatomical variations. For instance, if BMC were indeed more prevalent in females, as suggested by some studies on dry specimens (e.g., Hrdlička (1941) [4]), this could point to a possible developmental or hormonal influence that warrants further investigation.

Furthermore, the variability in prevalence values (ranging from 0.18% to 12.4%) found in Table 2 and Table 3 can be partly attributed to inconsistent methodology in case inclusion and denominator definitions. Some studies define prevalence by patient, others by condyle, while yet others mix symptomatic with incidental radiological findings. This creates significant heterogeneity and complicates inter-study comparisons. Finally, it is crucial to consider that the etiology of the BMC remains uncertain and multifactorial, involving congenital factors such as embryological persistence of septa, as in Nikolova et al. (2017) [8], as well as acquired influences including trauma, infection, or remodeling disturbances [10,13]. The lack of consensus on classification systems for BMC (i.e., based on morphological appearance, etiology, or clinical presentation) further hinders standardized reporting and comparative analysis.

In this context, our case offers an additional contribution to the literature, not only by reinforcing the predominance of unilateral, right-sided BMCs, but also by illustrating the value of high-resolution micro-CT in identifying subtle architectural patterns that may support developmental rather than acquired origins. Although the sex and age of the specimen were unknown, a limitation inherent to the study of dry anatomical material, our findings nonetheless emphasize the potential of advanced imaging and morphometric analysis to refine descriptive frameworks.

**Advancements in Imaging and Morphological Variations**

Historical anatomical studies provide a foundational framework, laying the groundwork for understanding the anatomical characteristics of BMCs. These earlier findings, such as those by Hrdlička (1941) [4] and Nikolova et al. (2017) [8], serve as critical reference points for contemporary CBCT-based research, which builds upon and expands this knowledge with greater diagnostic precision and broader epidemiological scope. Hrdlička described “double condyles” in 21 human jaws and one gorilla from the Smithsonian Institution’s collection, noting unilateral cases as more common and a slight female predominance. He also observed arthritic changes in the temporomandibular joint. Nikolova et al. analyzed 500 male dry jaws from Bulgaria and identified four unilateral BMCs (0.8%), with no bilateral cases and no evidence of trauma. These findings align with contemporary imaging-based data in terms of lateral predominance and rarity of bilateral forms.

Recent studies employing CBCT further refine our understanding. Yelken Kendirci et al. (2023) reported detecting mandibular condyles with multiple heads in 975 Turkish patients undergoing CBCT studies, finding BMCs in 5.84% (unilateral, 5.02%; bilateral, 0.82%) [23]. Likewise, they found the presence of a trifid mandibular condyle in two patients and a tetraphid mandibular condyle in one female patient. Regarding trifid mandibular condyles, Zoabi et al. (2022) [46] also described their finding in a female patient. Such observations underscore the improved sensitivity of CBCT in capturing anatomical complexity.

In some cases, a separate glenoid fossa is present for each of the two condylar heads, whereas in cases of traumatic origin, only a single glenoid fossa is observed. It has also been suggested that the differing orientations of the condylar heads may correspond to distinct underlying causes. When the condylar heads are aligned in the sagittal plane, a traumatic origin is typically suspected. In contrast, when the heads are oriented in the coronal plane, a developmental origin, such as the persistence of fibrous tissue septa, has been proposed [47]. Based on this, the present case of BMC may correspond to a developmental origin due to the orientation in the coronal plane.

Within this context, the radiodense appearance and trabecular continuity observed in our specimen are more consistent with a developmental variant rather than a neoplastic lesion such as an osteoma or osteochondroma. The absence of features typically associated with tumoral processes, such as defined margins, cortical disruption, or a cartilage cap, further supports this interpretation.

While CBCT has undeniably enhanced the morphological documentation of BMCs, our study introduces a novel dimension by applying high-resolution micro-CT to a dry mandible, allowing for precise quantification of internal bone architecture. We report detailed measurements such as trabecular thickness, separation, number, anisotropy, and connectivity density, parameters that are not typically obtainable through clinical imaging. This level of granularity enables structural comparisons between bifid and normal condyles within the same specimen, offering new insights into the biomechanical and developmental implications of this anatomical variant.

Since this study is the first of its kind, no data were found in the literature to contrast with the BMC. Previous studies have evaluated differences in trabecular bone microarchitecture between two edentulous regions of the mandible [48]. Therefore, further research should be conducted to assess the mandibular condyle with varying bone densities, both with and without pathology, using micro-CT scanning protocols. To our knowledge, this is the first report to provide such comprehensive microstructural profiling of a BMC using ex vivo micro-CT.

We propose classifying BMC into two types based on its etiopathogenesis, as described by Szentpétery et al. [47]:

**BMC Type I**: Developmental origin, characterized by two condylar heads oriented in the coronal plane and the presence of separate glenoid fossae.

**BMC Type II**: Traumatic origin, characterized by two condylar heads oriented in the sagittal plane and a single glenoid fossa.

**Statistical Interpretation Challenges**

Comparing data from dry bone studies and clinical imaging reveals that patient-based studies often report higher prevalence rates, which may reflect differences in methodological design, such as sample selection criteria, diagnostic imaging sensitivity, and detection thresholds. In particular, the widespread use of CBCT in clinical settings may contribute to more frequent incidental identification of anatomical variants like BMCs [49]. However, methodological heterogeneity complicates direct comparisons. A major issue is the inconsistent use of denominators; some studies calculate prevalence per patient, others per condyle, which affects reported values. Additionally, most studies fail to specify the distribution of BMCs by sex and side, limiting the ability to accurately determine prevalence.

This distinction between prevalence (used in Table 3) and proportion (used in Table 2) is critical. It is crucial to differentiate between prevalence (proportion of cases about a defined population, such as women) (Table 3) and proportion (distribution of observed cases between categories such as sex or sides) (Table 2). For example, in a hypothetical situation with 10 women and 90 men where there are 6 left unilateral BMCs, although the sex ratio is the same in men and women (50%, 3 BMCs in women/6 BMCs), the prevalence differs significantly (women: 30%; 3 left BMC of women/10 left condyles of women; 3.3%, three left BMC of men/90 left condyles of men). Misinterpretations like these emphasize the need for consistent definitions and transparent methodologies in anatomical epidemiology.

In this regard, while our case report does not contribute epidemiological prevalence data due to the lack of demographic context, it underscores the complementary value of anatomical specimen-based studies in refining descriptive parameters. Specifically, by delivering intra-individual quantitative comparisons between a bifid and a contralateral normal condyle within the same mandible, our findings help establish reference values for trabecular structure that could inform future radiological interpretations. Such anatomical benchmarking may prove particularly useful in validating or calibrating CBCT-based estimations where microstructural accuracy is limited.

**Biomechanical Implications and Bone Microarchitecture**

Beyond morphological characterization, recent work has begun to explore the biomechanical properties of BMCs, providing a deeper understanding that complements the statistical patterns presented in Table 2 and Table 3. While prevalence and distribution data highlight how frequently and in whom BMCs occur, bone quality studies provide insights into how these anatomical variants may functionally behave. This integrated perspective, combining epidemiological trends with microstructural assessments, enriches our interpretation of BMCs, especially in relation to their clinical relevance and potential association with temporomandibular disorders. Bone quality is influenced not only by mineral density and volume but also by the microarchitecture of trabecular bone [50,51,52].

In the case reported, concerning the quality of the bone tissue of the jaw, the cortical bone on the right side had a relatively large volume and represented 64.70% of the volume of interest. The trabecular bone surface was more significant compared to the cortical trabecular bone. The trabecular thickness was smaller with greater spacing, which may be characteristic of a less dense structure. On the left side, cortical bone represented 71.81% of the total tissue volume. The bone surface was greater, and the trabecular thickness was very high, suggesting a denser and more robust bone structure than the right side. Based on the results, the cortical bone on the left side had a larger volume and a higher percentage of the total volume than the right. The trabecular bone on the left side also showed a larger absolute volume but with differences in trabecular structure (larger spacing, smaller thickness than the right side). These differences in bone density and structure between the two sides may be relevant to evaluating bone conditions in cases of facial asymmetries, trismus, or joint pain [8].

Despite many BMC cases being identified through imaging, few studies have assessed bone microarchitecture. Previously, no studies had provided data on the quality and quantity of bone tissue in a BMC. This omission represents a significant limitation in the anatomical and functional understanding of this variant. Although several cases of BMC have been documented through radiographic or tomographic studies, most have focused on external morphology, the number of condylar heads, or their spatial orientation. However, the bone microarchitecture of these atypical condyles has not been characterized, making it difficult to identify potential differences in bone quality parameters, such as trabecular pattern, bone volume fraction, porosity, and the orientation of bone laminae, compared to a normal mandibular condyle.

Based on the results obtained in our study, and from a biomechanical perspective, the BMC exhibits a less dense bone architecture, with lower cortical volume, fewer and more widely spaced trabeculae, and overall reduced structural compactness. This suggests a structure that is less adapted to withstand mechanical stress, with potential functional implications for mandibular movement and the development of temporomandibular disorders. Moreover, the presence of a BMC could be associated with temporomandibular joint ankylosis [36].

Furthermore, micro-CT is a high-precision three-dimensional imaging technique that evaluates bone morphology and microarchitecture in ex vivo tissues [17], whether under normal, senescent, or pathological conditions [52]. This allows the cortical and trabecular bone to be analyzed without altering the jaw, as in a conventional histological technique [18].

## 4. Conclusions

This study offers a novel contribution to the anatomical characterization of bifid mandibular condyles (BMCs) by combining osteological observation with high-resolution micro-computed tomography. Through a direct comparison between a bifid condyle and its contralateral normal counterpart within the same specimen, we quantitatively assessed trabecular bone parameters including thickness, separation, number, and connectivity. These findings revealed distinct differences in bone microarchitecture that may reflect underlying biomechanical adaptations or developmental variations associated with the bifid morphology.

To our knowledge, this is one of the first studies to present such detailed internal structural data for a BMC, highlighting the potential of micro-CT imaging to expand our understanding beyond what is visible through conventional radiographic or gross anatomical methods. This approach not only enhances the descriptive anatomical framework for BMCs but also sets a foundation for future investigations into their functional implications, such as altered load distribution, joint degeneration, or potential surgical complications.

Although the literature review included in this manuscript contextualizes the rarity, morphological spectrum, and diagnostic trends of BMCs, our primary contribution lies in providing measurable structural insights that were rarely addressed in prior reports. In doing so, we advocate for the inclusion of microarchitectural analysis in future anatomical and clinical studies to improve the characterization, classification, and potential risk stratification of bifid condylar variants.

Future research directions should focus on expanding the sample size of structurally analyzed BMCs, integrating demographic and clinical data where available, and exploring possible correlations between internal morphology and clinical symptoms. Such efforts could contribute significantly to understanding the developmental origins and clinical relevance of this uncommon anatomical variation.

## Figures and Tables

**Table 1:** Histomorphometric measurements obtained by micro-CT.

		BV mm^3^	C.BV/C.TV %	BS mm^2^	Tb.Th µm	Tb.N mm^−1^	Tb.Sp µm
BMC-right	Cortical Trabecular	727.46 413.27	64.70 35.91	2191.93 3189.51	1281 721	504 497	648 805
MC-left	Cortical Trabecular	1291.68 444.63	71.81 31.21	3120.84 4239.31	1690 440	422 694	581 808

BMC: bifid mandibular condyle; MC: mandibular condyle; BV: bone volume; BV/TV: absolute bone volume; BS: bone surface; Tb.Th: trabecular thickness; Tb.N: trabecular number; Tb.Sp: trabecular separation.

**Table 2:** Distribution by side and sex of cases of bifid mandibular condyle reports in the literature (only cases since 2010 specifying the sex and the side of the anatomical variation were considered).

References	Male			Female		
	L	R	B	L	R	B
López-López et al. (2010) [24]	0	0	0	2	0	0
Sala-Pérez et al. (2010) [25]	0	3	0	0	2	1
Kamtane and Subramaniam (2011) [26]	0	0	0	0	1	0
De Melo et al. (2011) [12]	0	0	0	0	0	1
Katti et al. (2012) [27]	0	1	0	0	0	0
Tanner et al. (2012) [28]	0	0	1	0	0	0
Neves et al. (2013) [29]	0	1	0	1	2	0
Prol et al. (2017) [30]	0	0	0	0	0	1
Borrás-Ferreres et al. (2018) [9]	1	0	0	1	0	0
Phore et al. (2018) [31]	0	0	1	0	0	0
Miranda et al. (2019) [32]	0	1	0	0	0	0
Coclici et al. (2020) [33]	0	1	0	0	0	0
Rajashri et al. (2021) [34]	1	0	0	0	0	0
Çelik et al. (2022) [35]	0	0	1	0	0	0
Gupta et al. (2022) [22]	8	11	0	10	11	0
Michalski et al. (2022) [36]	1	0	0	0	0	0
Arumugam Venkatachalam Sargurunathan et al. (2023) [37]	1	0	0	0	0	0
Total	12	18	3	14	16	3

L: left; R: right; B: bilateral.

**Table 3:** Prevalence of the bifid mandibular condyle.

References	% (BMC/TM)	% (BC/TC)	Male (BCM/TMC)	Female (BCF/TFC)	Left (BCL/TLC)	Right (BCR/TRC)	Bilateral (BBMC/TM)
Menezes et al. (2008) [14]	0.18% (9/50,080)	0.11% (11/10,0160)	X% (3/X)	X% (8/X)	0.008% (4/50,080)	0.006% (3/50,080)	0.004% (2/50,080)
Balaji (2010) [38]	12.4% (15/21)	7.8% (19/242)	X% (7 + bilat) ^1^	X% (8 + bilat) ^1^	7.4% (9/121)	1.65% (2/121)	3.3% (4/121)
Miloglu et al. (2010) [39]	0.31% (32/10,200)	0.19% (40/20,400)	X% (15/X)	X%(17/X)	0.1% (10/10,200)	0.14% (14/10,200)	0.08% (8/10,200)
Sahman et al. (2011) [40]	0.52% (98/18,798)	0.33% (125/37,596)	X% (47 + bilat/16,242) ^1^	X% (51 + bilat/21,354) ^1^	0.18% (34/18,798)	0.2% (37/18,798)	0.14% (27/18,798)
Haghnegahdar et al. (2014) [41]	3.5% (35/1000)	1.9% (38/2000)	X% (12 + bilat/466) ^1^	X% (23 + bilat/1534) ^1^	2.4% (24/1000)	0.8% (8/1000)	0.3% (3/1000)
Khojastepour et al. (2015) [42]	4.5% (14/309)	2.7% (17/618)	3.6% (10/278)	2% (7/340)	1.9% (6/309)	1.6% (5/309)	0.97% (3/309)
Gündüz et al. (2015) [43]	1.6% (42/2634)	0.85% (45/5268)	0.72% (21/2910)	1% (24/2358)	0.8% (21/2634)	0.68% (18/2634)	0.11% (3/2634)
Gupta et al. (2022) [22]	4.7% (40/850)	2.3% (40/1700)	5.6% * (19/335)	4% * (21/515)	1.2% (10/850)	1.6% (14/850)	1% (8/850)

% = Prevalence; X = Not reported. % (BMC/TM) = Number of mandibles (or patients) with bifid condyle/total number of mandibles (or patients); % (BC/TC) = Total number of bifid condyles/total number of condyles; (BCM/TMC) = Total number of bifid condyles in men/total number of condyles in men; (BCF/TFC) = Total number of bifid condyles in women/total number of condyles in women; (BCL/TLC) = Total number of bifid condyles on the left side/total number of left condyles; (BCR/TRC) = Total number of bifid condyles on the right side/total number of right condyles; (BBMC/TM) = Number of mandibles with bilaterally bifid condyles/total number of mandibles. ^1^: Bilateral condyles were recorded by the authors as a single case instead of two condyles. Since the authors did not specify the sex distribution of the bilateral cases, it was not possible to include the missing condyle for each case for men and women in the table. * The number of men and women in the table and in the article text differ. We have used the values presented in the table.

## Data Availability

The raw data supporting the conclusions of this article will be made available by the authors on request.

## Abbreviations

The following abbreviations are used in this manuscript:

BMC	Bifid mandibular condyle
TMJ	Temporomandibular joint
micro-CT	Micro-computed tomography
